# LH pulse frequency and the emergence and growth of ovarian antral follicular waves in the ewe during the luteal phase of the estrous cycle

**DOI:** 10.1186/1477-7827-7-78

**Published:** 2009-07-28

**Authors:** Srinivas V Seekallu, Behzad M Toosi, Norman C Rawlings

**Affiliations:** 1 Department of Veterinary Biomedical Sciences, Western College of Veterinary Medicine, University of Saskatchewan, Saskatoon, SK, S7N 5B4, Canada

## Abstract

**Background:**

In the ewe, ovarian antral follicles emerge or grow from a pool of 2–3 mm follicles in a wave like pattern, reaching greater than or equal to 5 mm in diameter before regression or ovulation. There are 3 or 4 such follicular waves during each estrous cycle. Each wave is preceded by a peak in serum FSH concentrations. The role of pulsatile LH in ovarian antral follicular emergence and growth is unclear; therefore, the purpose of the present study was to further define this role.

**Methods:**

Ewes (n = 7) were given 200 ng of GnRH (IV) every hour for 96 h from Day 7 of the estrous cycle, to increase LH pulse frequency. Controls (n = 6) received saline. In a second study, ewes (n = 6) received subcutaneous progesterone-releasing implants for 10 days starting on Day 4 of the cycle, to decrease LH pulse frequency. Controls (n = 6) underwent sham surgery. Daily transrectal ovarian ultrasonography and blood sampling was performed on all ewes from the day of estrus to the day of ovulation at the end of the cycle of the study. At appropriate times, additional blood samples were taken every 12 minutes for 6 h and 36 min or 6 h in studies 1 and 2 respectively.

**Results:**

The largest follicle of the follicular wave growing when GnRH treatment started, grew to a larger diameter than the equivalent wave in control ewes (P < 0.05). Mean serum estradiol and progesterone concentrations were higher but mean serum FSH concentrations were lower during GnRH treatment compared to control ewes (P < 0.05). The increased serum concentrations of estradiol and progesterone, in GnRH treated ewes, suppressed a peak in serum concentrations of FSH, causing a follicular wave to be missed. Treatment with progesterone decreased the frequency of LH pulses but did not have any influence on serum FSH concentrations or follicular waves.

**Conclusion:**

We concluded that waves of ovarian follicular growth can occur at LH pulse frequencies lower than those seen in the luteal phase of the estrous cycle but frequencies seen in the follicular phase, when applied during the mid-luteal phase, in the presence of progesterone, do enhance follicular growth to resemble an ovulatory follicle, blocking the emergence of the next wave.

## Background

In the ewe, recent studies using transrectal ovarian ultrasonography have revealed a wave like pattern in the growth and regression of ovarian antral follicles. During the breeding season [1-4] and anestrus [5,6], 1–3 follicles emerge or continue to grow from a pool of small follicles (2–3 mm in diameter) every 3–5 days. These follicles reach diameters of ≥ 5 mm before ovulation or regression. Each follicular wave is preceded by a peak in serum concentrations of FSH lasting 3 to 4 days; this peak has been shown to be an essential trigger for the emergence of a follicular wave [1-3,6,7]. Prior to the use of ultrasonography for repetitive, non invasive monitoring of ovarian structures in the ewe, studies on the endocrine regulation of ovarian folliculogenesis were largely limited to single surgical or post mortem observation of the ovary. From such studies, it was concluded that the growth of ovarian antral follicles beyond 2 to 3 mm in diameter was largely dependent on FSH, with the final growth and maturation becoming LH dependent [8-11].

During the luteal phase of the estrous cycle in sheep, the frequency of secretion of pulses of LH is quite low, in the order of 1 to 2 pulses per 6 h [12]. This low frequency is maintained by the negative feedback effects of progesterone in concert with estradiol [13-15]. In the folicular phase, LH pulse frequency increases to ≥ 1 pulse per hour, once the inhibitory effects of progesterone are removed [16,17]. The reduced LH pulse frequency of the luteal phase is believed to hold final follicular growth and development in check while the enhanced pulse frequency of the follicular phase is believed to drive such maturation. In a recent study, using ewes with ovarian transplants and given a GnRH antagonist, antral follicles grew from 4.5 mm to 4.9 mm in diameter, over a period of 66 hours with only basal LH secretion. This change in size was not significant and was not enhanced by treatment with pulses of LH or constant infusion of a low dose of LH over a similar time period. However, increasing the dose of LH given by constant infusion gave a significant increase in follicle size (3.9 to 5.0 mm in diameter) [18]. These follicles luteinized but did not ovulate in response to hCG. When we looked for correlation between characteristics of follicular waves (eg; length of the growth phase, maximum follicle size, numbers of follicles in a wave etc) at various time points in the estrous cycle and the secretory patterns of LH at the same time points, there was no indication of significant associations [19,20]. However, when ewes were treated to create low serum progesterone concentrations from Day 4 after ovulation, LH pulse frequency increased and the largest follicles of the first wave of the cycle was still growing 9 days later [21].

To try and clearly define the role of pulsatile LH secretion in the regulation of follicular waves in the ewe, we did two experiments designed to alter LH pulse frequency over a wide range without affecting the FSH peaks that herald follicular waves. The objective of experiment 1 was to increase LH pulse frequency, during the midluteal phase of a cycle, to that seen in the follicular phase and the objective of experiment 2 was to suppress LH pulse frequency to a value less than that seen in the normal luteal phase. We hypothesized that altering LH pulse frequency over the range described would not influence any aspect of ovarian follicular waves.

## Methods

### Animals

Care and handling of experimental animals was done according to the Canadian Council on Animal Care's published guidelines. Sexually mature, clinically healthy, cycling (November–December), Western White Face (WWF) ewes were kept outdoors in sheltered paddocks (Saskatoon, SK, Canada; latitude: 52°10'N). Ewes were fed a maintenance diet of hay; cobalt iodized saltlicks and water were freely available. The WWF is a cross between the Columbia and Rambouillet breeds. Ewes were monitored daily for estrus with vasectomized crayon marker-harnessed rams.

### Ultrasound technique

Ovarian antral follicular dynamics were monitored in all ewes by transrectal ovarian ultrasonography (scanning) using a 7.5-MHz transducer stiffened with a hollow plastic rod and connected to a B-mode, real-time echo camera (Aloka SSD-900, Overseas Monitor, Richmond, BC, Canada). This technique has been validated for monitoring ovarian follicular dynamics and for CL detection in sheep [6,7]. All images were viewed at a magnification of × 1.5 with constant gain and focal point settings. Ovarian images were recorded (Panasonic AG 1978, Matsushita Electric, Mississauga, ON, Canada) on high-grade video tapes (Fuji S-VHS, ST-120 N, Fujifilm, Tokyo, Japan) for later examination. The relative position and dimension of follicles and luteal structures were also sketched on ovarian charts.

### Blood sampling

Blood samples (10 mL) taken daily were collected by jugular venipuncture into vacutainers (Becton Dickinson, Franklin Lakes, NJ, USA). Intensive blood sampling and injection of GnRH or saline was done via indwelling jugular catheters (5 ml/sample; vinyl tubing, 1.0 mm inside diameter × 1.5 mm outside diameter; SV70, Critchley Electrical Products Pty Ltd., Auburn, NSW, Australia). All samples were permitted to clot at room temperature for 18 to 24 h. Samples were then centrifuged for 10 min at 1500 × g, and serum was removed and kept at -20°C until assayed.

### Experimental design

#### Experiment 1

**Figure 1:**
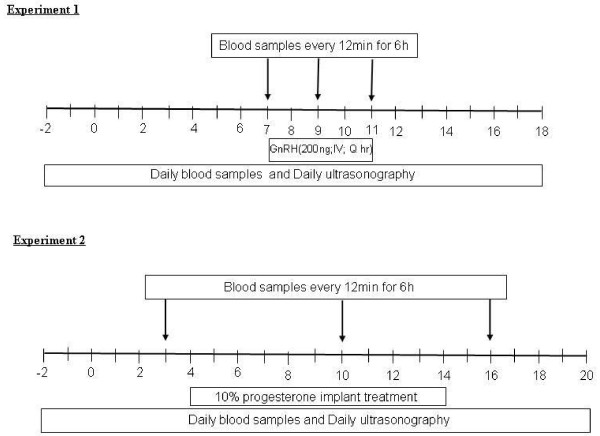
**Schematic representation of the experimental designs used in the present study**. In experiment 1, injections of GnRH (200 ng; IV) were given every hour for 96 h starting on Day 7 after ovulation (Day 0). In experiment 2, progesterone implants were inserted subcutaneously 4 days after ovulation and remained in place for 10 days. Control animals received saline or empty implants in experiment 1 and 2, respectively. Daily transrectal ovarian ultrasonography and blood sampling was performed on all ewes starting from the day when the ewe was marked with a vasectomized crayon-harnessed ram (estrus) until ovulation at the end of cycle. Blood samples were also taken every 12 minutes for 6 h (intensive bleeds) on Day 7, Day 9 and Day 11 after ovulation in experiment 1 and on Day 3, Day 10 and Day 16 in experiment 2, to characterize the pulsed secretion of LH and or FSH.

Daily transrectal ovarian ultrasonography and blood sampling was performed on all 13 randomly selected ewes (mean body weight of 82.73 ± 2.5 kg), starting from the day when they were initially marked by the vasectomized crayon-harnessed rams (estrus) until ovulation at the end of the cycle of study. Blood samples were taken daily at 12 noon, following ultrasound scanning. Starting at 8 AM on Day 7 after ovulation (Day 0), seven ewes were given injections of GnRH (200 ng; IV; in saline; Sigma Chemical Company, St. Louis, MO, USA; Figure 1) every hour for 96 h, with the last injection given at 8 AM on Day 11. The dose of GnRH, was designed to create LH pulses that were within a physiological range and was derived from dose trial studies. Six control ewes received injections of saline. Additional blood samples were taken every 12 minutes (intensive sampling), from 36 minutes before to 6 h after the GnRH injection given at 8 AM on Day 7, Day 9 and Day 11 after ovulation.

#### Experiment 2

Twelve randomly selected ewes (mean body weight of 79.04 ± 2.2 kg) were divided into two groups of six ewes each. Six ewes received subcutaneous silastic rubber implants (11 × 0.48 cm; 2 implants per ewe) containing 10% progesterone w/w (125 mg/implant; Sigma-Aldrich, Oakville, ON, Canada; [22]) on Day 4 after ovulation (Day 0; Figure 1); implants remained in place for 10 days. Six sham operated control ewes received no implants. To make the implants, liquid silastic rubber (A-101 medical grade silicone elastomer; Factor II Inc., Lakeside, AZ) was mixed with the steroid, and a curing catalyst was added (Catalyst; Factor II Inc.). The mixture was injected into Tygon tubing moulds (0.48 cm i.d.; Norton Plastics, Akron, OH). Once cured, the progesterone implants were removed from the moulds, and cut into 11 cm lengths. Implants were soaked in sterile saline, in a water bath, at 37°C for 48 h before insertion. Lidocaine hydrochloride (2%; Xylocaine; Astra Zeneca Canada Inc., Mississauga, ON, Canada) was used as a local anesthetic. A 1.5-cm incision was made in the axillary region with a scalpel, the implant was inserted using a trocar, and the incision was closed with wound clips (9-mm MikRon AUTOCLIP; Becton Dickinson Primary Care Diagnostics, Sparks, MD, USA). Each animal received one 11 cm implant in the left and right axillary regions, respectively. Daily transrectal ovarian ultrasonography and blood sampling was performed on the same schedule as described for experiment 1. Blood samples were also taken every 12 min for 6 h on Day 3, Day 10 and Day 16 after ovulation starting at 8 AM.

### Analysis of follicular data

A follicular wave consisted of a follicle or a group of follicles that emerged and grew from 2 or 3 mm in diameter to ≥ 5 mm (growth phase), before regressing to 2 or 3 mm in diameter (regression phase) or ovulation; time spent at ≥ 5 mm was regarded as the static phase [2,6]. The length of the growth, static and regression phases, growth rate and life span of the largest follicle growing in follicular waves were analyzed for the interovulatory interval studied. The lifespan of a large antral follicle is defined as the interval from its emergence at 2 or 3 mm to its regression back to 2 or 3 mm. If more than 1 follicle attained the same maximum size, the follicle that reached the maximum diameter first and/or remained at its maximum size for the longest period of time, was regarded as the largest follicle of the wave. The number of small (≥1 to ≤3 mm in diameter), medium (4 mm in diameter), and large sized follicles (≥ 5 mm in diameter) and maximum follicle diameter each day, as well as the number of follicular waves, were also analyzed for the interovulatory interval studied. Ovulation was detected with ultrasonography as the collapse of a large follicle that had been followed in its growth/static phase for several days. Follicular data were integrated for both ovaries of each ewe.

### Hormone analysis

Progesterone [15], estradiol [23], FSH [24] and LH [25] concentrations were measured in serum samples by validated RIA procedures. Steroid assays used antisera produced in our laboratory with standards obtained from the Sigma Chemical Company [15,23] and the reagents for the gonadotropin assays were obtained from NIDDK/NHPP. The assay sensitivities (defined as the lowest concentration of a hormone capable of significantly displacing radio-labeled hormone from the antibody) were: 0.03 ng/mL for progesterone, 1.0 pg/mL for estradiol and 0.1 ng/mL for FSH and LH. The ranges of standards were: 0.1 to 5 ng/mL, 1.0 to 100 pg/mL, 0.12 to 16.0 ng/mL, and 0.063 to 8.0 ng/mL for the progesterone, estradiol, FSH, and LH assays, respectively. A concentration equivalent to the sensitivity of the assay was assigned to serum samples with hormone concentrations lower than the assay sensitivity. Serum samples collected daily, throughout the experimental period, were analyzed for concentrations of progesterone, estradiol, and FSH. All serum samples collected every 12 minutes were analyzed for concentrations of LH; FSH was also measured in experiment 1.

For Experiments 1 and 2, the intra- and inter-assay coefficients of variation (CVs) were 11.4% and 9.6% or 7.1% and 13.7% for reference sera with mean progesterone concentrations of 0.26 or 1.17 ng/mL, respectively. The intra- and inter-assay CVs were 9.2% and 11.0% or 6.7% and 11.1% for reference sera with mean estradiol concentrations of 7.83 or 23.36 pg/mL, respectively. The intra- and inter-assay CVs were 4.8% and 7.2% or 7.9% and 11.8% for reference sera with mean LH concentrations of 0.41 or 2.77 ng/mL, respectively. For Experiment 1, The intra-assay CVs were 2.2% or 4.0% for reference sera with mean FSH concentrations of 1.57 or 3.68 ng/mL, respectively. For Experiment 2, the intra-assay CVs were 2.3% or 3.4% for reference sera with mean FSH concentrations of 0.86 or 4.04 ng/mL, respectively.

The PC-PULSAR program [26] was used to assess mean and basal serum FSH and LH concentrations as well as FSH and LH pulse frequency and amplitude in blood samples collected every 12 min for 6 h. The basal serum level ("smoothed series") was generated after the removal of short-term variation in hormone concentrations, including possible pulses. Standard deviation criteria (G and Baxter parameters) were used for pulse detection.

Peaks of FSH in blood samples taken daily were identified using cycle-detection software [27]. A fluctuation or cycle was defined as a progressive rise and fall in hormone concentrations that encapsulated a peak concentration (nadir-to-peak-to-nadir; [27]). Mean basal FSH concentrations were determined by averaging the lowest points between peaks (nadirs). Follicle stimulating hormone peak concentration was defined as the concentration of FSH observed at the apex of the FSH peak. Follicle stimulating hormone peak amplitude was defined as the difference between the FSH peak concentration and the nadir before the peak concentration.

### Statistical analyses

All data for hormone concentrations and ovarian follicles measured daily were normalized to the day of ovulation both for presentation and statistical analyses. Two-way repeated measures ANOVA (Sigma Stat 7 for Windows Version 2.03, 1997, SPSS Inc.; Chicago, IL, USA) was used to assess time trends and treatment effects on hormone concentrations and numbers of follicles in different size classes. Two-way repeated measures ANOVA was also used to assess differences in parameters of LH and FSH secretion in blood samples collected every 12 min for 6 h amongst treated and control ewes and between days of intensive sampling. Two-way repeated measures ANOVA was used to assess differences in characteristics of FSH peaks (i.e., FSH peak concentration and amplitude, FSH peak duration and basal FSH concentration) and ovarian follicles (i.e., length of growth, static and regression phases, life span and growth rate) amongst peaks/waves detected during the interovulatory interval studied and amongst the treated and control ewes. The t-test was used to compare the number of FSH peaks and follicular waves, interovulatory interval and ovulation rate amongst the treated and control ewes. If the main effects, or their interactions, were significant (P < 0.05), in the repeated measures ANOVA's, then Fisher's protected least significant difference (LSD) was used as a post-ANOVA test to detect differences between individual means (P < 0.05). Data are expressed as mean ± SEM.

## Results

### Experiment 1

#### Characteristics of serum LH concentrations

**Figure 2:**
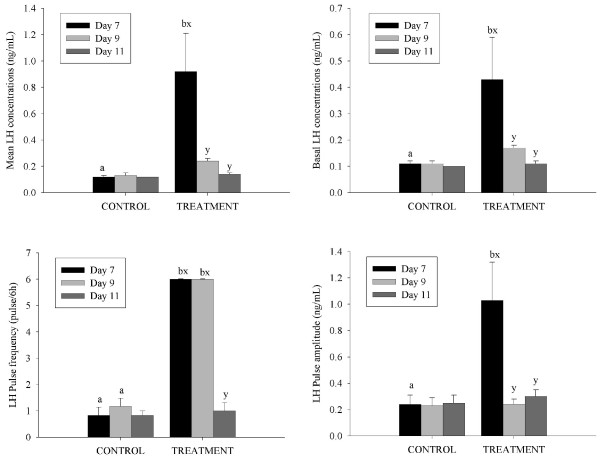
**The characteristics of pulsatile LH secretion (mean and basal serum LH concentrations and LH pulse amplitude and frequency; mean ± SEM) determined from serum samples collected every 12 min for 6 h on Day 7 (black bars), Day 9 (light gray bars) and Day 11 (dark grey bars) after ovulation in cyclic Western White Face ewes**. Starting on Day 7 after ovulation, ewes were treated with GnRH (200 ng; IV; treatment group) or saline (control group) every hour for 96 h. Letters (a-b) indicate differences between control ewes and ewes treated with GnRH (P < 0.001) within the respective intensive sampling period. Letters (x-y) indicate differences between the days of intensive blood sampling (P < 0.001) for control ewes or ewes treated with GnRH. [n = 6 (control), n = 7 (treatment)].

Ewes were given GnRH every hour from 8 AM on Day 7 after ovulation to 8 AM on Day 11 (96 h). It should be noted that on Day 11 blood sampling started 36 minutes prior to the last injection of GnRH and continued for 6 h after injection. During the periods of intensive blood sampling, each injection of GnRH was seen to cause an LH pulse. Based on the data from blood samples collected every 12 min for 6 h, treatment with GnRH resulted in an increased LH pulse frequency on Days 7 and 9 compared to control ewes (P < 0.05; Figure 2). Mean and basal serum LH concentrations and LH pulse amplitude however, were only greater in GnRH treated ewes and compared to control ewes on the first day of treatment (Day 7 after ovulation; P < 0.05; Figure 2).

#### Mean daily serum estradiol concentrations

**Figure 3:**
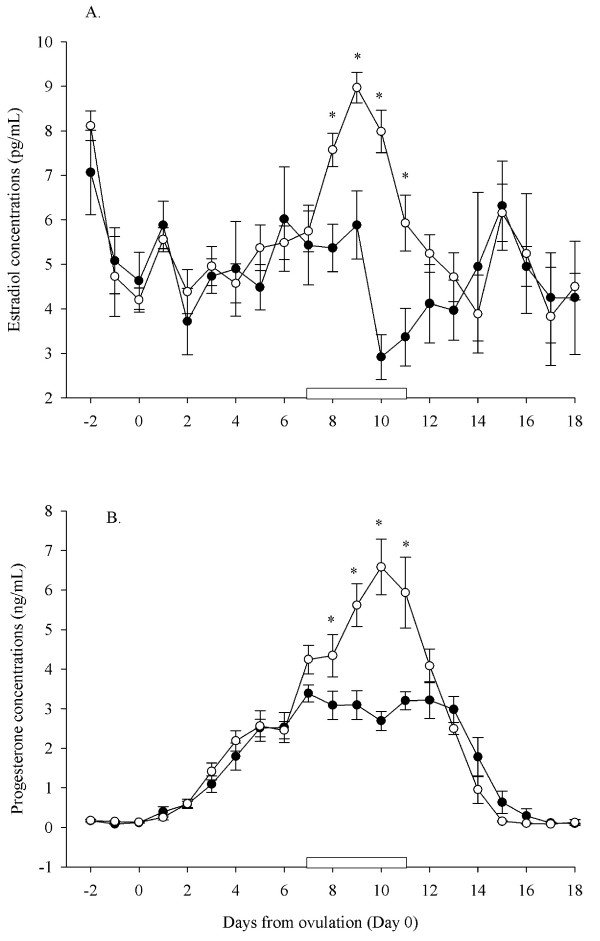
**Mean (± S.E.M.) daily serum estradiol (Panel A) and progesterone concentrations (Panel B) over the entire experimental period in cyclic Western White Face ewes treated every hour for 96 h, with GnRH (200 ng; IV; open circles; n = 7) or saline (black circles; n = 6), starting on Day 7 after ovulation (Day 7–11; indicated by an open rectangular box on the X-axis)**. Data were normalized to the day of ovulation (Day 0) in all ewes. Asterisks (*) indicate differences between ewes treated with GnRH and control ewes (P < 0.001).

During the period of treatment with GnRH or saline, serum estradiol concentrations were higher in ewes treated with GnRH compared to control ewes (7.24 ± 0.5 pg/mL vs. 4.59 ± 0.7 pg/mL; P < 0.001; Figure 3A). Comparison of individual means showed that mean serum estradiol concentrations were significantly higher in ewes treated with GnRH compared to control ewes on Days 8 to 11 after ovulation (Figure 3A).

#### Mean daily serum progesterone concentrations

During the period of treatment with GnRH or saline, serum progesterone concentrations were higher in ewes treated with GnRH compared to control ewes (5.35 ± 0.6 ng/mL vs. 3.09 ± 0.3 ng/mL; P < 0.001; Figure 3B). Comparison of individual means showed that mean serum progesterone concentrations were significantly higher in ewes treated with GnRH compared to control ewes on Days 8 to 11 after ovulation (Figure 3B).

#### Characteristics of serum FSH concentrations

**Figure 4:**
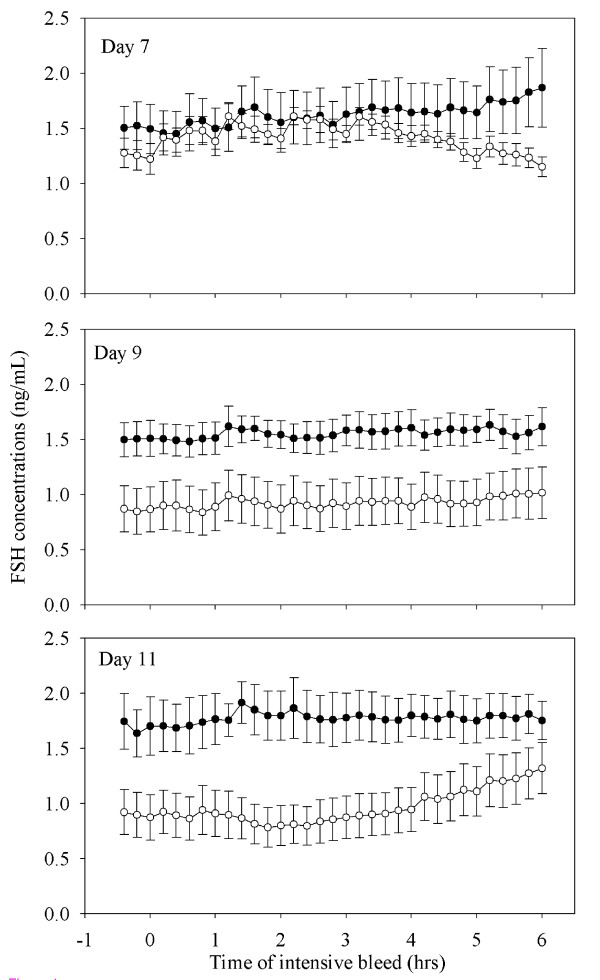
**Mean (± S.E.M.) serum FSH concentrations determined from serum samples collected every 12 min for 6 h on Day 7 (top panel), Day 9 (middle panel) and Day 11 (bottom panel) after ovulation in cyclic Western White Face ewes treated every hour for 96 h, with GnRH (200 ng; IV; open circles; n = 7) or saline (black circles; n = 6), starting on Day 7 after ovulation**.

Based on blood samples taken every 12 min for 6 hrs on Day 7, Day 9 and Day 11 after ovulation, FSH secretory profiles were found to be non pulsatile by the PC-PULSAR program [26] and no response to the injection of GnRH was seen (P > 0.05; Figure 4). In ewes treated with GnRH, mean serum FSH concentrations were lower on Days 9 and 11 compared to Day 7 after ovulation; concentrations on Day 9 and 11 were also lower than in control ewes (P < 0.05; Figure 4).

**Figure 5:**
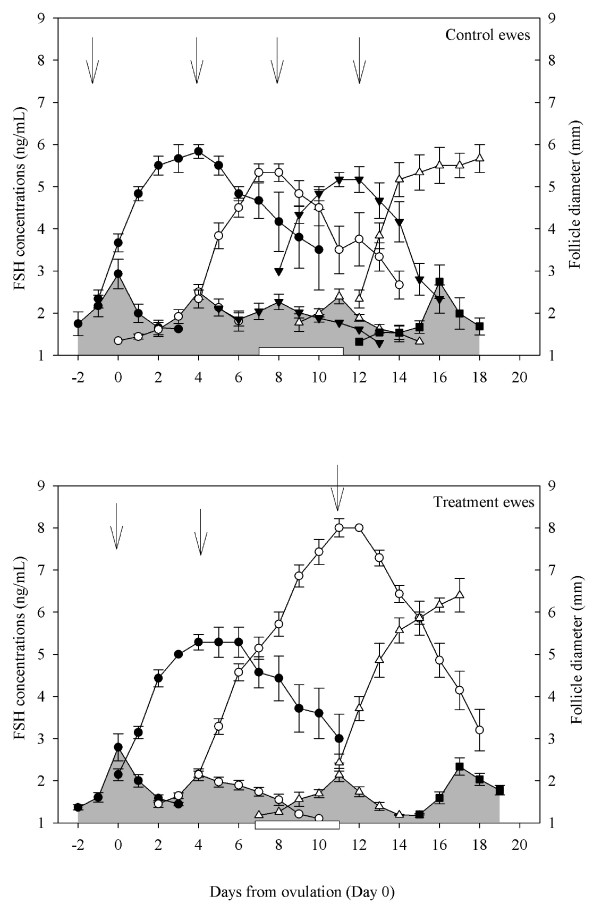
**Peaks in serum concentrations of FSH (outlined with shading) and their associated emerging follicular waves in cyclic Western White Face ewes treated every hour for 96 h, with GnRH (200 ng; IV; bottom panel; n = 7) or saline (top panel; n = 6), starting on Day 7 after ovulation (Day 7–11; indicated by an open rectangular box on the X-axis)**. Data were normalized to the day of ovulation (Day 0) in all ewes. Concentrations of FSH and follicle diameters are expressed as mean ± S.E.M. The average curves representing the growth, static and regression phases of all ewes in a group were normalized for each follicle wave to the mean day of wave emergence (indicated by the arrows; [36]). All FSH peaks for all ewes are shown normalized to the mean day of occurrence of the apex of the FSH peak for each wave. Although most follicular waves emerge at the zenith of the FSH peak, emergence can occur one day before or after the zenith of the FSH peak. For every FSH peak, serum concentration profiles were delimited by the encompassing nadirs of the FSH concentrations (hence the overlap of the data for adjacent peaks in some cases).

Based on blood samples collected daily, there were no differences in FSH peak concentrations and amplitude, basal FSH concentrations and FSH peak duration amongst control ewes and ewes treated with GnRH (P > 0.05; Figure 5). During the interovulatory interval studied, there were only 4.0 ± 0.0 FSH peaks in GnRH treated ewes compared to 5.0 ± 0.0 in control ewes (P < 0.05; Figure 5). In the interovulatory interval studied, the time from the 2^nd ^FSH peak (Day 4 in both ewes treated with GnRH and control ewes) to the next FSH peak (Day 11 in ewes treated with GnRH and Day 8 in control ewes) was longer in ewes treated with GnRH compared to control ewes (7.43 ± 0.3 vs. 3.83 ± 0.4 days; P < 0.05; Figure 5). The 3^rd ^FSH peak detected on Day 8 in control ewes was missing in ewes treated with GnRH (Figure 5).

#### Antral follicle development, ovulations and corpus luteum

During the interovulatory interval studied, we observed 4 follicular waves with corresponding FSH peaks in control ewes but only 3 follicular waves with corresponding FSH peaks in ewes given GnRH (P < 0.05; Figure 5). The time from the day of emergence of the 2^nd ^follicular wave (Day 4 in both groups) to emergence of the next follicular wave was longer in ewes treated with GnRH compared to control ewes (6.86 ± 0.4 vs. 4.00 ± 0.5 respectively; P < 0.05; Figure 5). The 3^rd ^follicular wave, emerging on Day 8 in control ewes, was missing in ewes treated with GnRH (Figure 5). The lifespan of follicles growing in the 2^nd ^follicular wave was longer in ewes treated with GnRH compared to control ewes (14.14 ± 0.3 vs 9.0 ± 0.8 days; P < 0.001; Figure 5). This was due to a prolonged growth phase in ewes given GnRH compared to control ewes (6.43 ± 0.4 vs 3.0 ± 0.3 days; P < 0.001; Figure 5). Interestingly, within GnRH treated ewes, the length of the growth phase of follicles growing in the 2^nd ^follicular wave was greater than for the first follicular wave (6.43 ± 0.4 vs 3.71 ± 0.4 days; P < 0.001; Figure 5) but not different from that of the final or ovulatory wave of the interovulatory interval (4.57 ± 0.9 days; P > 0.05; Figure 5). The mean duration of the interovulatory interval (17.29 ± 0.2 d vs. 17.67 ± 0.2 d; P > 0.05; Figure 5) and the ovulation rate (2.14 ± 0.3 vs. 1.67 ± 0.2; P > 0.05) did not differ among the GnRH treated and control ewes.

**Figure 6:**
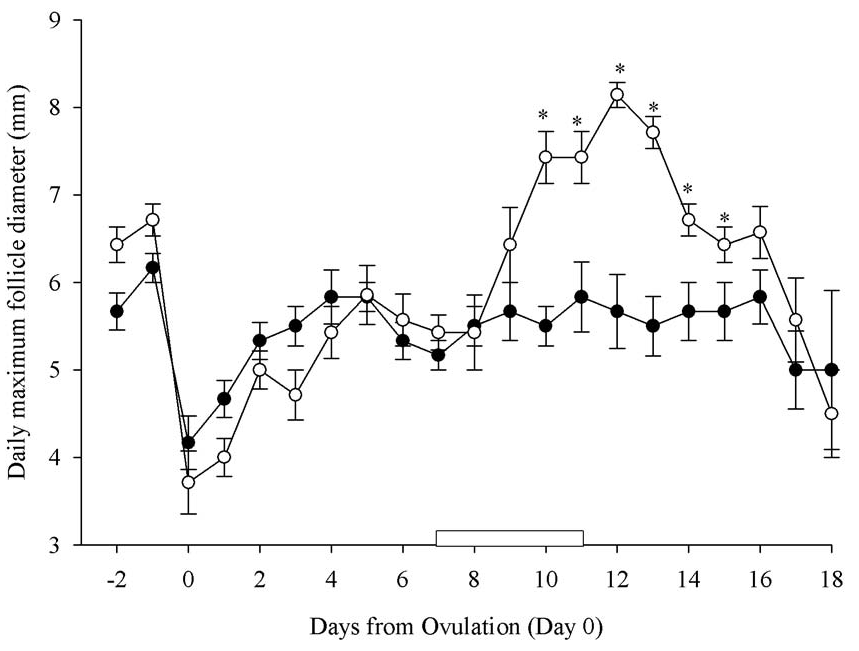
**Mean (± S.E.M.) daily maximum follicle diameter over the entire experimental period in cyclic Western White Face ewes treated every hour for 96 h, with GnRH (200 ng; IV; open circles; n = 7) or saline (black circles; n = 6), starting on Day 7 after ovulation (Day 7–11; indicated by an open rectangular box on the X-axis)**. Data were normalized to the day of ovulation (Day 0) in all ewes. Asterisks (*) indicate differences amongst ewes treated with GnRH and control ewes (P < 0.001).

During the interovulatory interval studied and as measured daily, there were no differences in the number of small (25.16 ± 1.8 vs 26.57 ± 1.5), medium (2.62 ± 0.7 vs 2.07 ± 0.6) or large sized follicles (2.25 ± 0.4 vs 2.10 ± 0.4) amongst the control ewes and the ewes treated with GnRH (P > 0.05). Maximum follicle diameter, measured on a daily basis, was greater in ewes given GnRH compared to control ewes during the period of treatment with GnRH (6.43 ± 0.3 mm vs. 5.56 ± 0.3 mm; P < 0.001; Figure 6). Comparison of individual means showed that maximum follicle diameter measured daily was significantly higher in ewes treated with GnRH compared to control ewes from 10 to 15 d after ovulation (P < 0.05; Figure 6). Maximum diameter of the corpus luteum, measured on a daily basis, did not differ between the control ewes and ewes treated with GnRH during the treatment period (10.73 ± 0.4 vs 10.86 ± 0.8 mm; P > 0.05).

### Experiment 2

#### Mean daily serum progesterone concentrations

**Figure 7:**
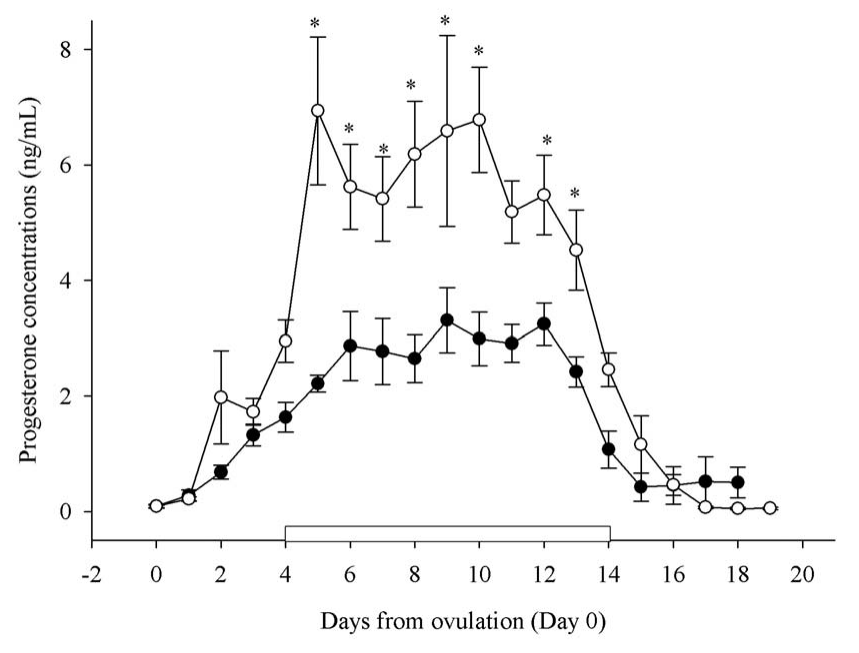
**Mean (± S.E.M.) daily serum progesterone concentrations over the entire experimental period in cyclic Western White Face ewes treated for 10 d, from Day 4 after ovulation (Day 4–14; indicated by an open rectangular box on the X-axis) with silastic rubber implants (s.c) containing 10% progesterone (open circles; n = 6) and sham operated control ewes (black circles; n = 6)**. Data were normalized to the day of ovulation (Day 0) in all ewes. Asterisks (*) indicate differences between ewes treated with GnRH and control ewes (P < 0.001).

Serum progesterone concentrations were higher in ewes treated with implants releasing progesterone compared to control ewes (5.28 ± 0.8 ng/mL vs. 2.55 ± 0.4 ng/mL; P < 0.001; Figure 7) during the period of treatment with progesterone releasing implants. Comparison of individual means showed that mean serum progesterone concentrations were significantly higher in ewes treated with implants compared to control ewes on Days 5 to 13 after ovulation (P < 0.05; Figure 7).

#### Characteristics of serum LH concentrations

**Figure 8:**
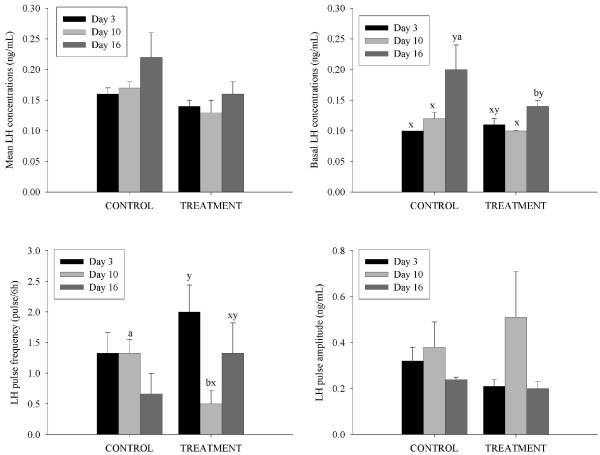
**The characteristics of pulsatile LH secretion (mean and basal serum LH concentrations and LH pulse amplitude and frequency; mean ± SEM) determined from serum samples collected every 12 min for 6 h on Day 3 (black bars), Day 10 (light gray bars) and Day 16 (dark grey bars) after ovulation in cyclic Western White Face ewes**. Ewes were treated for 10 d, from Day 4 after ovulation with silastic rubber implants (s.c) containing 10% progesterone (open circles; n = 6) and sham operated control ewes (black circles; n = 6). Letters (a-b) indicate differences between control ewes and ewes treated with progesterone releasing implants (P < 0.001) within respective intensive sampling period. Letters (x-y) indicate differences between the intensive blood sampling days (P < 0.001) for control ewes or ewes treated with progesterone releasing implants. [n = 6 (control), n = 6 (treatment)].

Ewes were given progesterone releasing implants on Day 4 after ovulation; implants were removed on Day 14. Based on data from blood samples collected every 12 min for 6 hrs, treatment with progesterone releasing implants decreased LH pulse frequency on day 10 compared to day 3 after ovulation and on Day 10, pulse frequency was lower than in control ewes, (P < 0.05; Figure 8). Basal serum LH concentrations were increased on Day 16 after ovulation in both groups of ewes but concentrations in control ewes on day 16 exceeded those treated with progesterone implants (P < 0.05; Figure 8).

#### Characteristics of serum FSH concentrations

**Figure 9:**
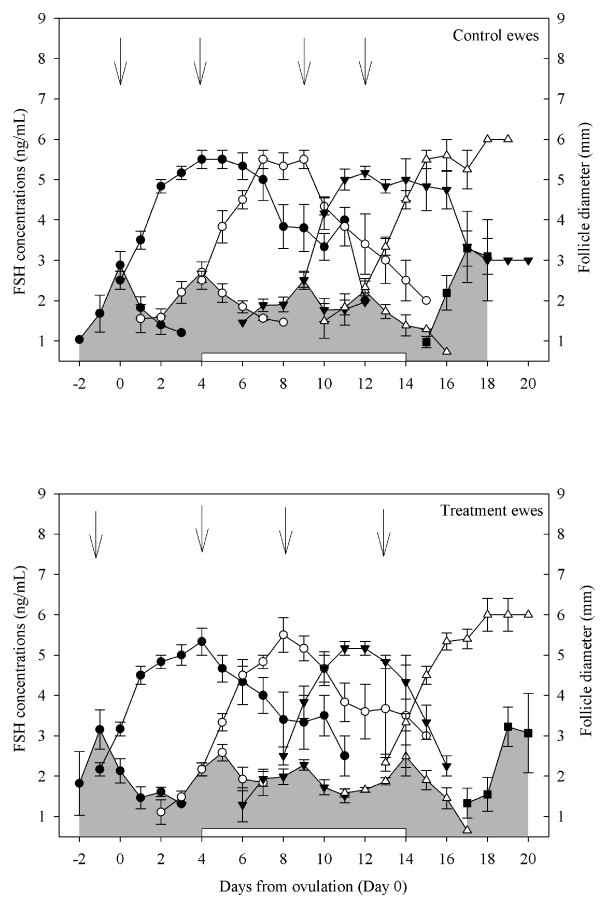
**Peaks in serum concentrations of FSH (outlined with shading) and their associated emerging follicle waves in cyclic Western White Face ewes treated for 10 d, from Day 4 after ovulation (Day 4–14; indicated by an open rectangular box on the X-axis) with silastic rubber implants (s.c) containing 10% progesterone (bottom panel; n = 6) and sham operated control ewes (top panel; n = 6). Data were normalized to the day of ovulation (Day 0) in all ewes**. Concentrations of FSH and follicle diameters are expressed as mean ± S.E.M. The average curves representing the growth, static and regression phases of all ewes in a group were normalized for each follicle wave to the mean day of wave emergence (indicated by the arrows; [38]). All FSH peaks for all ewes are shown normalized to the mean day of occurrence of the apex of the FSH peak for each wave. Although most follicular waves emerge at the zenith of the FSH peak, emergence can occur one day before or after the zenith of the FSH peak. For every FSH peak, serum concentration profiles were delimited by the encompassing nadirs of the FSH concentrations (hence the overlap of the data for adjacent peaks in some cases).

Based on blood samples collected daily, there were no differences in the number of FSH peaks, FSH peak concentration, amplitude and duration, or basal FSH concentration amongst control ewes and ewes treated with progesterone releasing implants (P > 0.05; Figure 9).

#### Antral follicle development and ovulations

The number of follicle waves emerging per animal did not differ amongst implant treated and control ewes (P > 0.05; Figure 9) during the interovulatory interval studied. All implant treated and control ewes had four follicular waves with corresponding FSH peaks. There were no differences in growth characteristics (growth, static, and regressing phase, growth rate and lifespan of the largest follicle in a wave) of follicular waves amongst implant treated ewes and control ewes (P > 0.05; Figure 9). The mean duration of the interovulatory interval (19.33 ± 0.6 d vs. 18.0 ± 0.4 d; P > 0.05; Figure 9) and the ovulation rate (2.33 ± 0.3 vs. 1.83 ± 0.2; P > 0.05) did not differ among the implant treated and control ewes. During the interovulatory interval and as measured daily, there were no differences in the number of small (22.77 ± 1.3 vs 21.95 ± 1.5), medium (1.46 ± 0.4 vs 1.58 ± 0.5) or large follicles (1.70 ± 0.3 vs 1.68 ± 0.4) or the daily maximum follicle diameter (5.61 ± 0.4 vs 5.17 ± 0.4 mm) amongst control ewes and implant treated ewes (P > 0.05).

#### Mean daily serum estradiol concentrations

Mean serum estradiol concentrations did not differ amongst progesterone treated and control ewes during the period of treatment with progesterone releasing implants (5.50 ± 1.0 pg/mL vs 4.72 ± 1.4 pg/mL; P > 0.05).

## Discussion

In experiment 1 of the present study, GnRH was given to ewes in the luteal phase of the cycle when LH pulse frequency was very minimal. The treatment created LH pulses with a frequency similar to the follicular phase of an estrous cycle [14,17]. The amplitude of the induced pulses on the first day of treatment (Day 7 after ovulation) was about four fold greater than that seen in control ewes but amplitude decreased to control values by Day 9 after ovulation and remained at that value until the end of the treatment (Day 11). Based on serum samples taken every 12 min, FSH secretion was non-pulsatile and showed no response to GnRH. This is not unexpected as FSH secretion is largely constitutive and does not occur in response to GnRH pulses [28-30]. It is interesting that creation of an LH pulse frequency, analogous to the follicular phase, in the present ewes, during the luteal phase of their cycle, increased follicular growth and estradiol secretion compared to control ewes. The effect on maximum follicle size was not significant until Day 10 after ovulation or after 3 days of GnRH treatment. The initial increase in LH pulse amplitude in response to GnRH had disappeared by Day 10 after ovulation. A significant increase in serum concentration of estradiol did occur by Day 8 but was sustained until Day 11. LH has been shown to stimulate estradiol production by way of receptors on the theca cells and later, as follicles mature, directly on the granulosa cells themselves [17,31,32]. These effects on antral follicle growth and function, even in a mileu of high concentrations of progesterone, in experiment 1 of the present study, would appear to have occurred largely due to the increase in LH pulse frequency. This contention is reinforced by the fact that serum concentrations of FSH declined over the period of GnRH treatment. The significant increase in basal serum concentrations of LH on Day 16 after ovulation in experiment 1 of the present study may reflect luteolysis and removal of progesterone negative feedback on basal LH secretion [33].

The large antral follicles of wave 2 of the cycle, that were exposed to the GnRH induced increase in LH pulse frequency, grew to a larger size than the equivalent follicles in the control ewes or even the follicles in wave 1 of GnRH treated ewes. The follicles of wave 2 in GnRH treated ewes grew and functioned in terms of estradiol production, like ovulatory follicles growing at the end of a cycle even in a mileu of high serum concentrations of progesterone. The follicles of wave 2 in GnRH treated ewes were not persistent but had a similar growth phase to ovulatory follicles in the control ewes but then regressed. It is intriguing that a follicular wave reminiscent of an ovulatory wave can be induced in the luteal phase of the cycle by increasing LH pulse frequency to that seen during the follicular phase. Clearly the presence of the serum concentrations of progesterone of the luteal phase does not directly inhibit a follicle from growing to ovulatory sizes when exposed to a significant increase in LH pulse frequency. However, the presence of progesterone did prevent the endocrine cascade that precedes and causes ovulation in the follicular phase of a cycle [14]. Preovulatory follicles at the end of the cycle in ewes tend to be somewhat larger and more estrogenic than the largest follicles seen in other follicular waves in the cycle [34]. However, the differences are small and rather variable. In the normal follicular phase in the ewe, LH pulse frequency is enhanced, compared to the luteal phase, but amplitude is actually reduced [33]. This again emphasizes the role of increased LH pulse frequency in the final growth and development of ovulatory follicles in the ewe.

In experiment 1, FSH secretion was suppressed during the period of GnRH treatment, compared to control ewes. In contrast to control ewes, there was no FSH peak at Day 8 after ovulation, on the second day of treatment, in GnRH treated ewes. This resulted in the loss of a follicular wave. We assume that the increased serum concentrations of estradiol and progesterone caused by GnRH treatment suppressed FSH secretion and blocked the peak in serum concentrations of FSH. Inhibin is also important in the negative feedback regulation of FSH secretion in the ewe but inhibin was not measured in the present study [9,10]. The existence of follicular dominance in the ewe, or the ability of secretory products of a large follicle to suppress the emergence and growth of other follicles, has been debated in the ewe [35]. Some form of limited dominance has been suggested [35]. In previous studies using subcutaneous implants releasing estradiol and progesterone, we have shown that supraphysiological serum concentrations of estradiol and progesterone suppressed FSH peaks [15,22,36]. We regard the doubling of serum concentrations of estradiol and progesterone, seen in GnRH treated ewes in the present study, as supraphysiological [22,36].

In those previous studies, when the amplitude of FSH peaks were decreased, follicular waves ceased but discernable FSH peaks continued to occur at the expected frequency [22,36]; such peaks appear to continue in ovariectomized ewes [37]. When we have created FSH peaks in cyclic ewes during the interwave interval by injections of ovine FSH (oFSH), follicular waves were induced even if the oFSH was given during the growth phase of a follicular wave resulting from an endogenously generated FSH peak [38]. In addition, FSH peaks created by injection of oFSH did not disrupt the normal rhythm of waves [38]. Injections of oFSH created physiological peaks in serum concentrations of FSH [36,38]. The studies above pose questions regarding the existence of antral follicular dominance in the ewe and suggest that the rhythm of FSH peaks in the ewe are not governed by changes in feedback regulation by secretory products of the large antral follicles in follicular waves but perhaps some yet unknown endogenous rhythm. It is intriguing that following the missed FSH peak and follicular wave, in GnRH treated ewes in the present study, the next FSH peak and emergence of a wave occurred on Day 11 at the same time as in control ewes. In other words, in GnRH treated ewes, an FSH peak was skipped but this did not perturb the overall rhythm of FSH peaks and follicular waves. However, it did appear that GnRH (LH) treatment enhanced the growth and estradiol production of the largest follicle of wave 2 of the interovulatory interval suppressing the next expected FSH peak and follicular wave. Based on follicle growth and serum concentrations of estradiol, it could be argued that this induced dominance appeared to wane prior to the FSH peak and follicular wave on Day 11.

In experiment 1 of the present study, serum concentrations of progesterone doubled in response to GnRH treatment. Progesterone secretion in the ewe is pulsatile with each pulse driven by a pulse of LH secretion [32]. Clearly enhancing LH pulse frequency from the minimal values of the luteal phase to values similar to the follicular phase enhanced the ability of the corpus luteum to secrete progesterone.

In experiment 2 of the present study, ewes were given progesterone-releasing implants during the luteal phase of an estrous cycle. The resulting serum progesterone concentrations were twice those seen in control ewes. Progesterone treatment decreased the frequency of secretion of LH pulses to less than half that seen in control ewes. The frequency of LH pulses is at a minimum during the luteal phase of the cycle in normal ewes and at a maximum of = 1 pulse per hour during the follicular phase [12,14,16,33]. The combined negative feedback effects of progesterone and estradiol are known to powerfully suppress LH pulse frequency [12,14-16]. The significant suppression of LH pulse frequency by progesterone, in the present study, to below that expected in a normal luteal phase, did not have any effect on the emergence, growth and regression of follicles growing in follicular waves. Serum concentrations of FSH were not significantly affected. In the cyclic ewe, the negative feedback effects of progesterone on FSH secretion are not as marked or as consistent as for LH [39].

In the cyclic ewe, follicular waves emerge every 3 to 5 days [2,4,40]. Each wave is heralded by a peak in serum concentrations of FSH and these peaks are essential triggers for the emergence of each wave [1-3,7]. However, when the secretory patterns of LH were correlated with the characteristics of follicular waves, both during the period from metestrus to the early luteal phase or during the mid to late luteal period of the estrous cycle, no consistent and major regulatory interactions were indicated [19,20]. Ewes in these two studies would have experienced frequencies of pulsed LH secretion ranging from the low frequencies of the mid to late luteal phase to the higher frequencies of the early luteal phase of the cycle. The characteristics of follicular waves studied included interwave interval, numbers of follicles in a wave, maximum follicle diameter, follicle lifespan subdivided into its phase, as well as the growth and regression rates of follicles [19,20].

In a recent study [18], using ewes with ovarian transplants and given a GnRH antagonist, antral follicles grew from 4.5 mm to 4.9 mm in diameter, over a period of 66 h with only basal LH secretion. This change in size was not significant and was not enhanced by treatment with pulses of LH or constant infusion of a low dose of LH over a similar time period. However, increasing the dose of LH given by constant infusion gave a significant increase in follicle size (3.9 to 5.0 mm in diameter) [18]. These follicles luteinized but did not ovulate in response to hCG. Suppression of the frequency of the pulsed secretion of LH in the present study, in intact cyclic ewes, to less than half of the frequency of the luteal phase of the estrous cycle, was consistent with normal follicular wave emergence and growth. These findings indicate that in the cyclic ewe only very low serum concentrations of LH, with very few pulses, are required to support ovarian follicular wave emergence and growth.

In conclusion, decreasing LH pulse frequency significantly, to values lower than the minimal frequencies seen in ewes in the luteal phase of an estrous cycle, did not affect any aspect of the growth and regression of follicular waves. Creating an LH pulse frequency, typical of the follicular phase, in ewes in the luteal phase of a cycle enhanced follicle growth and serum concentrations of estradiol and progesterone. Follicular waves growing in the luteal phase of a cycle in the ewe, in the presence of progesterone, can grow and function like ovulatory follicles growing in the follicular phase of the cycle, if exposed to an LH pulse frequency similar to the follicular phase. In GnRH treated ewes, enhanced secretion of estradiol and progesterone suppressed secretion of FSH peaks, blocking the emergence of a follicular wave. Loss of a follicular wave did not affect the timing of the next follicular wave when compared to control ewes.

## Competing interests

The authors declare that they have no competing interests.

## Authors' contributions

SVS, BMT were involved in the design of the study, performed the research and participated in discussion of the results. SVS drafted the manuscript. NCR was responsible for the conception, design, supervision and discussion of the study, as well as drafting and critical revision of the manuscript. All authors have read and approved the manuscript.
